# Dual process model of farmers’ mindfulness and sustainable economic behavior: Mediating role of mental health and emotional labor

**DOI:** 10.3389/fpsyt.2022.979979

**Published:** 2022-09-14

**Authors:** Yue Qiu, Yumeng Zhang, Meihang Liu

**Affiliations:** Department of Education, Liaoning Petrochemical University, Fushun, China

**Keywords:** mindfulness, mental health, emotional labor, sustainable economic behavior, farmers health stigma

## Abstract

Mindful awareness of our interconnection with the natural environment could help to redeem our lost environmentally entrenched identity and help us to act more sustainably, concluding the predictable gaps between mindfulness and sustainable behavior. We propose more precisely that mindful attentiveness may be essential to establishing sustainable economic behavior through understanding emotional labor and enhanced mental health. Likewise, with an ever-rising concern related to mental health and emotional labor, recent industrialization and commoditization of agricultural products have stressed the need for mindfulness, and causing sustainable economic behavior of farmers that is imminent. Hence, the study will not only explore the connection between mindfulness and sustainable economic behavior, but there is a need to examine the mediating role of emotional labor and the mental health of farmers in China. The study selected the farmers because mindful awareness, emotional labor, and mental health of a farmer can significantly contribute to sustainable economic behavior and bring a connection with the natural environment. The data of 358 responses were analyzed using SPSS-AMOS. The results revealed that mindfulness, mental health, and emotional labor have a significant connection with the sustainable economic behavior of farmers in China. The results also indicated that mental health and emotional labor mediate between mindfulness and sustainable economic behavior. The results set the tone for the policy-makers to create awareness among all the stakeholders about the importance of mindfulness to help farmers manage their emotional labor and mental health for better, sustainable performance outcomes.

## Introduction

The growth of business firms and the increase in economic activities may cause different forms of environmental pollution and adversely impact the stakeholders’ social welfare. The agriculture industry is no exception in this case (1). Usually, it is considered that the agricultural sector is associated with the growth of natural resources and develops a clean economic sector favorable for the environment and the general public. However, it is one side of the coin; even though it is linked to the growth of natural resources but may threaten the country’s development in the upcoming time. For instance, it may adversely impact the environment and social welfare of the public with the overuse of technologies, energy sources, chemicals, pesticides, fertilizers, and many other ingenious ways of increasing the production of crops, forests, and livestock (2). Employees’ mindfulness is the ability to be fully present, aware of where they are and what they are doing, and not unduly overwhelmed or reactive by what is happening around them; being mindful means paying attention to what a person is physically experiencing with the senses of mental state with thoughts and emotions. The mindful people can better focus, understand, and change the firm’s influences on the environment and social well-being to develop sustainable economic behavior (3). Likewise, one must understand that due to the recent worldwide economic crunch, there has been an unprecedented rise in emotional labor for professionals and the agriculture sector stakeholders. They are pushed to make ends meet, which somehow makes them indulge in actions that negatively affect the sustainable ecosystem of the environment.

A country not only needs to achieve higher economic growth like an improved position in the international market, foreign exchange earnings, increase in government revenues, and increase in the total production level of the country and employment rate, but they also need to sustain the once achieved higher growth rate (4). For sustainable economic development, the country should make progress in such a manner as it may not damage the environment, and the progress should be meant to meet the present needs of the public without compromising on the needs of the future generation. The main attributes of sustainable economic development are reducing pollution, increasing quality of life, and efficient use of natural resources (5). Sustainable economic development is contributed by individual business organizations operating in all economic sectors. The sustainable economic performance of business organizations depends on the personnel as these are the human resources who operate the business from establishment, management, leadership, production, and marketing. The organizational personnel, by themselves or through learning and training classes, have the attitude and tendency to participate in or take initiatives to establish the three pillars of sustainable economic performance: social, environmental, and financial performance. Employee attitudes and tendencies are termed sustainable economic behavior (6).

Therefore, this study examines the relationship between mindfulness, emotional labor, and mental health in developing sustainable economic behavior within agriculture firms in China. China is an emerging country with a higher middle-income economy and a newly industrialized developing country. It is the second-largest world economy in the nominal gross domestic product (GDP), whereas, in purchasing parity, it is the second-largest economy. The economy is estimated to attain a GDP of $19.91 trillion in 2022, which shows an 8.1% GDP growth rate (7). The Chinese economy has three main sectors: agriculture, industry, and service. According to the 2020 statistics, agricultural and allied industries made an added value of 16,690 billion yuan, which accounts for 16.47% of GDP. About 23.6% of the workforce in the Chinese agriculture system is employed. Though the country makes agricultural imports like soybeans, rubber, cotton, barley, and oils made from palm kernels and soybeans, the agricultural sector enables the country to increase total exports (8). According to 2019 statistics, the Chinese exports of agricultural products were USD 64.83 billion. Moreover, China is a major global livestock producer; it produces 50% of pigs, 20% of goats and chickens, and 15% of buffalos in the world inventory. As the Chinese agriculture sector is of great economic importance to the country, many farmers or ranchers have applied heavy technologies, chemicals, pesticides, and fertilizer to grow production and increase profits. These initiatives contaminate water, soil, turf, other vegetation, and the natural atmosphere and damage living creatures on land, water surface, and air (9). So, China holds the potential for setting trends related to sustainable behaviors of individuals across industries (i.e., agro-firms, to be specific). Therefore, exploring the phenomena of mindfulness and sustainable outcomes in the Chinese context is imperative.

Conversely, China is considered the biggest emitter of pollution emissions globally. It has been making rapid progress in all its economic sectors. However, suppose the pollution keeps increasing at a given rate. In that case, their economic progress may come into jeopardy, as consistent performance, a good working environment, an abundance of natural resources, and healthy and active humans are required affected by pollution. In China, like all other sectors, the agriculture sector has also been causing health and natural resources damaging consequences (10). To lead the country toward sustainable economic development, the agriculture sector must pay attention to sustainable economic behaviors. Hence this study has conceived the following objectives: (1) To investigate and analyze mindfulness, emotional labor, and mental impacts on sustainable economic behavior; (2) to investigate and analyze the impacts of mindfulness on emotional labor and mental health; (3) to analyze and investigate the role of emotional labor and mental health between mindfulness and sustainable economic behavior.

The study has a significant contribution to the literature. First, previous studies largely remained unable to investigate the relationship between sustainable mindfulness and its impact on sustainable economic performance. Second, in existing literature, no study analyses the role of mindfulness, emotional labor, and mental health in developing sustainable economic behavior. This study removes this literary gap by analyzing the impacts of mindfulness, emotional labor, and mental health’s role on sustainable economic behavior. Third, in the past, very few studies have been conducted to check the mediating role of emotional labor and mental health between mindfulness and sustainable economic behavior. This article removes the literary gap by analyzing emotional labor and mental health as mediators between mindfulness and sustainable economic behavior. Fourth, this pollution is because of the agriculture sector and its threats to economic development in China. Likewise, empirical investigations are scarce on measuring the enablers of sustainable economic performance across the agriculture sector. This article makes a distinction for analyzing the influences of mindfulness, emotional labor, and mental health on developing sustainable economic behavior in employees at farms in China.

## Literature review

The article investigates the impact of mindfulness, mental health, and emotional labor on the sustainable economic behavior of farmers in China. The article also examines the mediating impact of mental health and emotional labor among mindfulness and sustainable economic behavior. Sustainable economic behavior is the employees thinking that the firms must operate their activities in such a manner that they do not create any environmental or social issues, and the employees themselves must participate in ecologically friendly and socially friendly initiatives, which leads to sustainable economic performance. The sustainable economic behavior of the employees depends on their mentality and emotions. If the employees have mindfulness, they can have the mentality and emotions favorable to develop sustainable economic behavior (11, 12). The relationship between mindfulness, emotional labor, and mental health in developing sustainable economic behavior can be found in many places in the existing literature. Different authors have presented different views about the relationship between mindfulness, emotional labor, and mental health in developing sustainable economic behavior. This study sheds light on past authors’ opinions regarding mindfulness, emotional labor, and mental health in developing sustainable economic behavior and their relationship to establish an exact hypothesis.

In any economic sector, employees must be mindful to effectively observe, manage, and perform business functions to achieve the desired economic outcomes without negatively impacting the environment and public welfare. The agriculture sector is no exception, but this sector is more sensitive and requires more attention from farmers as it arranges for the basic needs of humans, fulfills the economic resources required, and has excellent interaction with the environment. If the farmers are mindful of whether the agriculture practices are linked to crops, forests, or livestock breeding, they can develop sustainable behavior (13). Likewise, it has been examined mindfulness’s impact on employees’ sustainable economic behavior (14). The study reveals that the firm personnel having high mindfulness usually remain conscious of aspects negatively affecting the sustainable performance of their businesses, which in turn helps them achieve sustainable performance goals. They develop sustainable economic attitudes and behavior to achieve sustainable economic development.

Similarly, this study examined the influences of mindfulness and sustainable consumption behavior (15). This study was conducted through a systematic analysis of the previous articles published in the considered time frame. Finally, seven studies were selected out of 32 studies under analysis. The reviewed studies used quantitative and qualitative approaches to examine the relationship of mindfulness with sustainable consumption behavior. The study shows that the employees who remain mindful while operating their functions within the organizations have learned the negative influences of the resources they utilize on the environment and their consequences. Therefore, the literature has observed that workers and employees with meaningful mindfulness usually remain in a better position to help their firms perform sustainably. After having environmental awareness, they prefer to utilize eco-logical friendly resources, energy-efficient technology, and renewable energy. Thus, mindfulness positively contributes to sustainable consumption behavior. The above-stated literature helps to establish the following hypothesis:

***H1:** Mindfulness has a positive relation to sustainable economic behavior.*

Emotional labor refers to managing emotions and emotional expressions to be consistent with the expectations about appropriate emotional expression within an occupation. Workers are explicitly expected to control their emotions when interacting with clients, coworkers, and managers. The suppression of emotions is felt but not expressed, including self-observation and decision-making about expressing emotion, whether or not it is genuinely felt. The same is applicable and fruitful in the case of farmers in agriculture (16). Likewise, previous studies have shown interest in exploring the relationship between emotional labor felt by farmers and agricultural workers and its association with sustainable attitudes, behaviors, and performance outcomes (17). The producers must consider the well-being of stakeholders and cultivate positive relationships with them to manage farming activities sustainably (18). Farmers who effectively control their emotions are better able to communicate with stakeholders and make decisions that are neutral and in the best interests of those stakeholders. Farmers employ sustainable economic practices as a result of their high emotional labor.

In an academic article on the triple labor framework for sustainable development initiatives, King (19) investigates emotional labor and sustainable economic behavior. The article is based on an ethnographic case study of initiatives for the alternative food system in Chiapas, Mexico analyzing three types of labor: organizational, physical, and emotional labor, and checks how these forms help develop sustainable economic behavior and achieve sustainable economic development. The study reveals that emotional labor assists the organizational personnel in the food production industry in having deep and trust-based connections with the customers. This connection helps them to have timely and reliable information from the customers about food products and other business matters and use that information in sustainable development initiatives. So, emotional labor positively develops sustainable economic development. Based on the reviewed literature, we put the following hypothesis:

***H2:** Emotional labor has a positive relation to sustainable economic behavior.*

In any workplace, whether the firms belong to the agriculture, industrial, or service sector, the workers must have the mental and physical health to perform their job functions efficiently. Only if the workers have good mental health will they be able to understand the working conditions and develop an atmosphere conducive to sustainable economic development (20). The study by Brouwers (21) examines the role of workers’ mental health role in adopting sustainable economic behavior. The study highlights that the workers’ mental health determines their cognitive abilities like observation, critical analysis, reasoning, planning, abstract thinking, complex idea comprehension, evaluation, decision making, and learning from experience. Workers with these capabilities can help implement sustainable economic development policies with sustainable behavior on their part. The study by Kruize et al. (22) is another investigation of the worker’s mental health role in developing sustainable economic development.

The study proclaims that when the workers of a firm have good mental health, they can better analyze the situation around them and will promptly reach the bottlenecks, hindering the sustainable goals of a firm. Hence, workers with managed mental health can facilitate sustainable economic behavior. The given economic downturn, it is obvious to expect that most of the workers already suffer from social, economic, and psychological distress (23). While the sound mental health of the personnel helps them fight against any form of distress and helps them overcome them, remain active, focus on the job functions, and perform ecologically. It confirms a positive link between workers’ mental health and sustainable economic behavior. Based on the past literary arguments, we may say:

***H3:** Mental health has a positive relation to sustainable economic behavior.*

A research article was written by Johnson and Park (24) to integrate the relationship between mindfulness through mindfulness training and emotional labor in the case of tourism and hospitality employees. This research article is based on the literature on mindfulness, training, emotional labor, burnout, and work engagement. The study reveals that when the real work at the workplace increases, it becomes an overburden for the employees, and they are not given much relaxation from work. Because of the overburden and consistent work engagement, the employee’s emotions got exhausted. Moreover, the employees are pressured with the threat that they may lose their job if they do not abide by the firms’ specific regulations and due to some uncertain event. In the firms where the employees are provided with mindfulness training, they learn how to manage their emotions and practice them during the job. A study by Ma et al. (25) claims that employees need to control their emotions at work. They must practice the feelings and actions that the circumstance at the time demands. As a result, people must continuously alter their emotions in response to situational changes. It is feasible when workers are vigilant and present at the moment at work, observing the environment and responding to changes in it. It clarifies that employees can engage in emotional labor if they have awareness. The study demonstrates the need for people to control their emotions at work. They must practice the feelings and behaviors that the circumstances at the time demand. As a result, people must continuously alter their expressions of emotions in response to changes in circumstances. It is likely to happen only when the workers are vigilant and fully present in the workplace, observing the situation, and reacting to changes. It clarifies that employees can have the ability of emotional labor if they have mindfulness (26). Based on the reviewed literature, we establish the following hypothesis:

***H4:** Mindfulness has a positive relation to emotional labor.*

Mindfulness is defined as one’s processing of internal stimuli. At the same time, the Cognitive psychologist’s definition of mindfulness focuses primarily on one’s processing of external stimuli. The study of Enkema et al. (27) identifies the relationship between mindfulness and mental health outcomes with the help of intensive longitudinal methods. The systematic literature review technique was applied, and articles were collected with the consideration of mindfulness measures or mindfulness interventions and by keeping in mind that with the adoption of intensive longitudinal methods for the assessment of mindfulness or mental health outcomes. The study revealed that mindfulness has a positive association with mental health. A mindful employee remains alert and adjusts to the circumstances’ fluctuation to meet their goals. They are less likely to accept any stress from emotional or financial distress. So, they enjoy mental health and can better concentrate on their work.

The study presented by De Cieri et al. (28) also investigates the farmer’s sustainable economic conduct and employees’ mental health. According to the study, a person’s mental health affects their cognitive and physical capabilities and ability to make decisions and actions. Farmers in good mental health, usually free of stress, emotional anguish, and financial problems, can make more wise decisions and have superior cognitive capabilities and natural physical talents. As a result, they can engage in sustainable economic behavior because they can assess how their actions and coworkers affect the environment and society and take steps to lessen those effects. The study analyses the influences of dispositional mindfulness on individuals’ mental health in education (29). The study results confirm that dispositional mindfulness negatively affects mental health issues. Dispositional mindfulness encourages decentering and self-acceptance, while decentering and self-acceptance help reduces mental health problems. Based on the past literary arguments, we may put the following hypothesis:

***H5:** Mindfulness has a positive relation to mental health.*

The empirical research of Nguyen et al. (30) analyses the relationship between mindfulness, emotional labor, and sustainable economic behavior. For emotional labor, the skills like endurance, patience, steadfastness, observation, analytical skills, the skill to make a practical choice, and management ability are required. The employees who have mindfulness by nature, learn from experience, and develop mindfulness from training, have achieved proficiency in all these skills. In this way, the employees’ mindfulness enhances the ability of emotional labor. When the employees are watchful and have the skills to modify their emotions according to the circumstance, they can have sustainable competitive advantages like effectively performing the assigned duties, removing problems, and finding ways through creativity. Hence emotional labor mediates between mindfulness and sustainable economic behavior. The study conducted by Salgado (31) highlights that when employees are mindful, they pay attention to their surroundings, consider the circumstances that led to them, and look for positive aspects. When they accept the changes and allow their emotions to adjust appropriately, they acquire the skills for emotional labor. For making sustainable business judgments, employees not only need to have a controlled mind but must have a heart full of managed emotions. Therefore, emotional labor as a result of mindfulness encourages workers to adopt sustainable economic behavior. The study presented by Hur et al. (32) also states that whenever firms have workers with emotional intelligence, it helps them not only manage their emotions but instead helps them in assessing the emotional aspects of the environment they work in; such organizations outperform other competing firms. This study proposed that:

***H6:** Emotional labor is a significant mediator between mindfulness and sustainable economic behavior.*

Burzler et al. (33) wrote an academic article investigating the relationship between mindfulness, mental health, and sustainable economic behavior. The employees having mindfulness remain alert, work actively, and change themselves with the situation change to achieve their goals. They are less likely to succumb to any stress brought on by emotional or financial hardships, which helps them maintain good mental health and focus more effectively at work. When employees are active, alert, watchful, and have a healthy mind to think, concentrate, and process, it becomes feasible to implement sustainable economic development goals because employees can acquire sustainable behavior. Hence, a healthy mind builds a link between mindfulness and sustainable economic behavior. In a literary workout, Carsley et al. (34) numerate the interrelationship between mindfulness, mental health, and sustainable economic behavior.

Through proper utilization of mindfulness, employees remain in a better position to keep their minds in check and not let them succumb to the pressure exerted by the distressing aspects of their personal and professional lives. It keeps the mind in practice and healthy. On the other hand, a healthy mind helps understand the significance of sustainable development and the ways to acquire it. Moreover, for considering and implementing sustainable initiatives, the management or operational department employees must have a sound mind that can comprehend the situation and explore new ways to maintain social and environmental performance along with economic progress (35). Therefore, it can be said:

***H7.** Mental health is a significant mediator between mindfulness and sustainable economic behavior.*

## Research methodology

It is explanatory research that employs a quantitative data-gathering strategy. The research investigates the impact of mindfulness, mental health, and emotional labor on the sustainable economic behavior of farmers. Also, it examines the mediating impact of mental health and emotional labor among mindfulness and sustainable economic behavior of farmers in China. The study selected the farmers as the unit of analysis and gathered the responses using questionnaires. The measurement item scales are adopted from past literature. The variable mindfulness has six items taken from the article of Zhang et al. (36). The variable emotional labor has a 4-item scale taken from the study of Lee and Chelladurai (37). Moreover, the variable mental health has a 5-item scale taken from Berwick et al. (38). Finally, the variable sustainable economic behavior has a 4-item scale and is taken from Tommasetti (39). These measurements are given in Table 1.

**TABLE 1:** Variables and measurements.

Items	Statements	Sources
**Mindfulness**		
MFN1	“I am aware that my emotions during harvesting can influence my thinking and behavior.”	(35)
MFN2	“When something unexpected happens during harvesting, I am aware of my emotional state.”	
MFN3	“When something during harvesting doesn’t go well, I am aware of my inner frustration and restlessness.”	
MFN4	“When the situation changes during the harvesting, I am aware of the thoughts and ideas that flashed across my mind.”	
MFN5	“When the harvesting process is beyond my expectations, I am aware of my physical reactions and changes.	
MFN6	“During harvesting, I can be immediately aware of my emotional changes.”	
**Emotional labor**		
EL1	“I always know my athletes’ emotions.”	(36)
EL2	“I am a good observer of athletes’ emotions.”	
EL3	“I am sensitive to the feelings and emotions of athletes.	
EL4	“I always know whether or not I am happy.”	
**Mental health**		
MH1	“Have you been a very nervous person?”	(37)
MH2	“Have you felt calm and peaceful?	
MH3	“Have you felt downhearted and blue?	
MH4	“Have you been a happy person?	
MH5	“Have you felt so down in the dumps that nothing could cheer you up?”	
**Sustainable economic behavior**		
SEB1	“Adopting sustainable practices is a good idea.”	(38)
SEB2	“Adopting sustainable practices is not a wise idea.”	
SEB3	“I like knowing that a farmer adopts sustainable practices.”	
SEB4	Farmers that pursue sustainability adopt appropriate behavior.	

There are two sections of the questionnaire. The respondent’s demographic information was obtained from the first part. In the second portion, we offered questions about each of our variables. The response to each question item was obtained using a 5-point Likert scale. The non-probability purposive sampling approach was used to acquire data. The questionnaire in this study was designed on a 5-point Likert scale. This form of questionnaire is said to help gather data from a large population in a trustworthy manner. Indeed, a survey-based questionnaire is vital for collecting data from respondents, since it is simple to disseminate and collect the questionnaire for analysis. As a result, this study used the same data-gathering strategy.

The scale questions were collected with careful attention and face validity. However, the common method bias (CMB) has been removed by adding a time delay, increasing the physical separation of items, or adding a cover story to deemphasize any association between the independent and dependent variables (40). This study has selected farmers as the respondents of the study. The farmers are chosen based on purposive sampling because only those with a wide range of arable land are selected. Eighty-six percent of the farmers were male, and only 14% were female, and out of those 14%, 6% were those with no son or husband who died early and managed the farms by themselves. Approximately, 63% of farmers were intermediate, 20% were undergraduates, and the rest were not qualified or had failed to answer this question. The surveys were sent to the selected farmers using personal visits. Ministry of Agriculture and Rural Affairs collects the list of farmers with a wide range of arable land.

**FIGURE 1:**
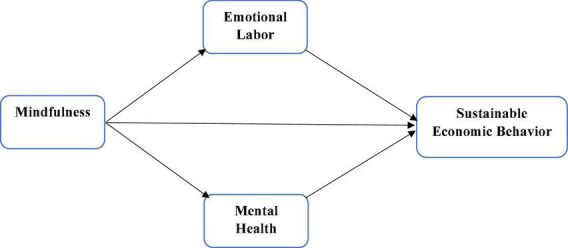
Theoretical framework.

The researchers have selected the top 5,000 farmers from the list as the total population. According to the Morgan sample size criteria, the sample size of the selected population is 357. Thus, the researchers sent 620 questionnaires to the farmers but only 358 valid surveys. These selected surveys have a 57.58% response rate. In addition, this study has taken one predictor such as mindfulness (MFN). In contrast, the study has taken two mediating variables such as emotional labor (EL) and mental health (MH), and the article has used only one dependent variable named sustainable economic behavior (SEB). These variables and their relationships are shown in Figure 1.

**FIGURE 2:**
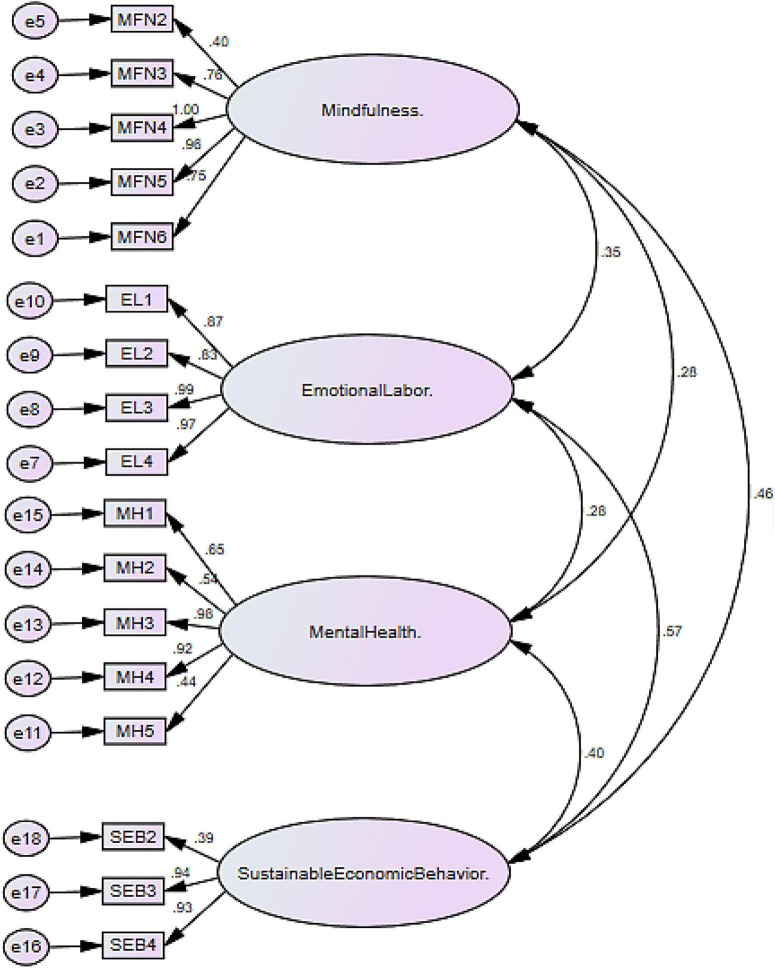
Measurement model assessment.

The study used the SPSS-AMOS to check the items and variables’ reliability and validity and also check the association among variables (see Figure 2). The applied tool deals effectively with large and small data sets and manages complex models (41). In addition, this tool assesses the validity and reliability using the measurement model and the association among variables using the structural model. In an assessment of the measurement model, convergent validity, discriminant validity, and reliability have been tested. The convergent validity has been examined with the help of maximum shared variance (MSV), the average squared shared variance (ASV), factor loadings, and average variance extracted (AVE). The standard criteria for MSV and ASV are that the values should be smaller than AVE (42, 43), while the standard criteria for AVE and factor loading are that the values should be more than 0.50 and 0.40 (44, 45), respectively. The face validity and content validity have been examined with the help of AVE and factor loadings even if you change the survey questionnaire to the native language. In addition, the reliability has been examined using composite reliability (CR), and the standard criteria are the value must be higher than 0.70 (45) and ignore Cronbach’s Alpha for this purpose as suggested in Refs. (44, 46).

Moreover, the discriminant validity shows the construct validity and has been investigated using Fornell Larcker, and the standard criteria for Fornell Larcker are that the first value must be more significant than the other values in the same column (47, 48). In addition, the study also investigated the model’s good fitness with the help of the Tucker–Lewis index (TLI), root means, the square error of approximation (RMSEA), standardized root mean square residual (SRMR), the goodness of fit index (GFI), adjusted goodness of fit index (AGFI), and the comparative fit index (CFI). The standard criteria for RMSEA are that the values should be lower than 0.05 (42), while the standard criteria for SRMR are that the values should be less than 0.09, the standard criteria for GFI and AGFI are that the values should be more than 0.95 and 0.80, respectively (49, 50). Moreover, the standard criteria for TLI and CFI are that the values should be more significant than 0.90 (51, 52). In addition, the association among variables has been tested using a structural model. The standard criteria for the direction of the association is the sign linked with beta values. If the sign is positive, then positive relationships between independent and dependent variables and vice versa. In addition, the standard criteria for significance are that the t-statistics should be higher than 1.64, and the probability values should be less than 0.05 (43, 53). These techniques are used in the upcoming section of the study.

## Research findings

The results section shows the examination of item correlation called convergent validity. The convergent validity has been examined with the help of MSV, ASV, factor loadings, and AVE. The standard criteria for MSV and ASV are that the values should be smaller than AVE (43, 52), while the standard criteria for AVE and factor loading are that the values should be more than 0.50 and 0.40, respectively (43). The results exposed that the factor loadings values are more significant than 0.40, AVE values are more prominent than 0.50, and MSV and AVE values are less than AVE values (43). These results indicated a high correlation among items and valid convergent validity. In addition, the results also show the examination of reliability using CR, and the standard criteria are that the values must be higher than 0.70 (43). The results revealed that the CR values were higher than 0.70 and indicated good reliability. These results are shown in Table 2.

**TABLE 2:** Convergent validity.

Constructs	Items	Loadings	CR	AVE	MSV	ASV
Mindfulness	MFN6	0.749	0.845	0.543	0.158	0.105
	MFN5	0.963				
	MFN4	0.999				
	MFN3	0.757				
	MFN2	0.404				
Emotional labor	EL4	0.974	0.893	0.643	0.211	0.139
	EL3	0.993				
	EL2	0.830				
	EL1	0.874				
Mental health	MH5	0.438	0.957	0.847	0.321	0.174
	MH4	0.920				
	MH3	0.983				
	MH2	0.539				
	MH1	0.647				
Sustainable economic behavior	SEB4	0.933	0.824	0.635	0.321	0.230
	SEB3	0.941				
	SEB2	0.394				

MNF, mindfulness; EL, emotional labor; MH, mental health; SEB, sustainable economic behavior.

In addition, the results section also shows the correlation among variables called discriminant validity. The discriminant validity has been investigated using Fornell Larcker, and the standard criteria for Fornell Larcker are that the first value must be more significant than the other values in the same column. The results exposed that the first value is larger than the rest and exposed low correlation among variables and valid discriminant validity (43, 48). These results are shown in Table 3.

**TABLE 3:** Discriminant validity.

	MH	MFN	EL	SEB
MH	0.737			
MFN	0.284	0.802		
EL	0.275	0.354	0.920	
SEB	0.398	0.459	0.567	0.797

MNF, mindfulness; EL, emotional labor; MH, mental health; SEB, sustainable economic behavior.

Moreover, the study also investigated the model’s good fitness with the help of TLI, RMSEA, and CFI. The standard criteria for RMSEA are that the values should be lower than 0.05, while the standard criteria for TLI and CFI are that the values should be more significant than 0.90 (52). The results revealed that the RMSEA value is lower than 0.05, and the TLI and CFI values are higher than 0.90 (52). Moreover, the results also exposed that the SRMR value is less than 0.09, the GFI value is more than 0.95 and the AGFI value is more than 0.80 (52). These values exposed that the model is a good fit. These results are shown in Table 4.

**TABLE 4:** Model fit indices.

Selected indices	Result	Acceptable level of fit
TLI	0.987	TLI > 0.90
CFI	0.965	CFI > 0.90
RMSEA	0.010	RMSEA < 0.05 good; 0.05 to 0.10 acceptable
GFI	0.971	GFI > 0.95
SRMR	0.022	SRMR < 0.09
AGFI	0.869	AGFI > 0.80

TLI, Tucker–Lewis coefficient; CFI, comparative fit index; RMSEA, root mean square error of approximation; GFI, goodness fix index; SRMR, standardized root mean squared residual; AGFI, adjusted goodness of FFIT index.

**FIGURE 3:**
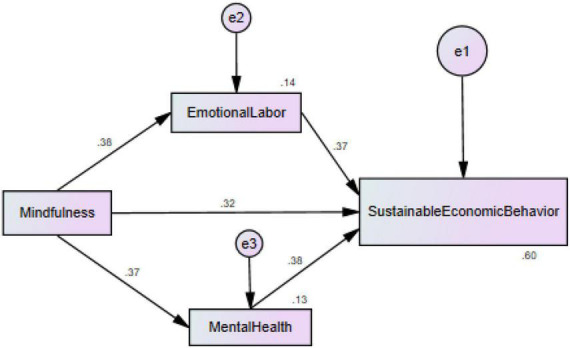
Structural model assessment.

The results of the structural model (Figure 3) revealed that mindfulness, mental health, and emotional labor have a positive linkage with the sustainable economic behavior of farmers in China because the beta values have a positive sign and accept H1, H2, and H3. The relationships among mindfulness, mental health, emotional labor, and sustainable economic behavior are significant because the t-statistics values are more prominent than 1.64, and probability values are lower than 0.05 (43, 54). Moreover, the results exposed that if 1% rise in mindfulness, sustainable economic behavior will also rise by 0.323% and vice versa. Furthermore, the results exposed that if a 1% rise in mental health, sustainable economic behavior will also rise by 0.383% and vice versa. Additionally, the results exposed that if 1% rise in emotional labor, sustainable economic behavior will also rise by 0.366% and vice versa. In addition, the results also revealed that mindfulness positively links mental health and emotional labor because the beta values have a positive sign and accept H4 and H5. The relationships among mindfulness, mental health, and emotional labor are significant because the t-statistics values are more extensive than 1.64, and probability values are lower than 0.05. Moreover, the results exposed that if 1% rise in mindfulness, emotional labor will also rise by 0.380% and vice versa. Similarly, the results exposed that if 1% rise in mindfulness, mental health will also rise by 0.366% and vice versa. These associations are given in Table 5.

**TABLE 5:** Path analysis.

Relationships			Std. Beta	Beta	SE.	CR.	*P*
EL	< —	MFN	0.380	0.461	0.059	7.747	***
MH	< —	MFN	0.366	0.334	0.045	7.422	***
SEB	< —	MFN	0.323	0.310	0.037	8.427	***
SEB	< —	MH	0.383	0.402	0.038	10.691	***
SEB	< —	EL	0.366	0.290	0.028	10.170	***

MNF, mindfulness; EL, emotional labor; MH, mental health; SEB, sustainable economic behavior. ***Represent relationship and significance between variables.

Moreover, the results also indicated that mental health significantly and positively mediates mindfulness and sustainable economic behavior and acceptance H6 because the collective effect of mental health and mindfulness is positive. Moreover, the results also indicated that emotional labor significantly and positively mediates mindfulness and sustainable economic behavior and accept H7 because the collective effect of emotional labor and mindfulness is positive. These associations are given in Table 6.

**TABLE 6:** Indirect effects.

Relationships	Beta	Lower limit	Upper limit
MFN > MH > SEB	0.141	0.302	1.992
MFN > EL > SEB	0.141	0.187	1.726
Total indirect effect	0.282	0.982	2.776

MNF, mindfulness; EL, emotional labor; MH, mental health; SEB, sustainable economic behavior.

## Discussions

The paper investigates the impact of mindfulness, mental health, and emotional labor on the sustainable economic behavior of farmers in China. The article also examines the mediating impact of mental health and emotional labor among mindfulness and sustainable economic behavior. The results showed that mindfulness has a positive relation to sustainable economic behavior. These results are in line with the study by Chan (42), which shows that in the agriculture sector, the farmers have many duties like preparation of land, planting, harvesting, breeding animals, feeding, collecting food products, performing manual labor, making a choice, and purchasing farming equipment, leading a team of farm workers, marketing of crops, forests, and animal-based products. Therefore, every decision of farmers not only holds the potential to affect their agricultural performance but can also negatively affect the environment. If the farmers are mindful, they can have better decisions and sustainable behavior. These results are also supported by Wamsler et al. (44), who examined farmers’ mindfulness and its impacts on farmers’ sustainable behavior. The study posits that if the farmers have high-level mindfulness, they not only want economic progress in the present time but try to sustain it. They keep an eye on the functioning of their farms and analyze the impacts of farm activities on the quality of the environment and stakeholders’ health. If they find any harmful strains, they try to remove them. Hence, farmers’ mindfulness leads to sustainable economic behavior.

The study results revealed that emotional labor positively relates to sustainable economic behavior. These results are supported by Sarraf (47), which highlights the emotional labor’s role in shaping sustainable behavior. The study proclaims that farmers have to perform strict physical duties in agriculture. Performing physical activities within the boundaries of a specific timetable can exert fatigue and exhaust the farmer’s emotions, which may lead them to make wrong decisions. However, if the farmers practice emotional labor, they restrain from wrongdoing and apply sustainable economic behavior. Hence, farmers with high emotional labor can develop sustainable economic behavior. These results are also in line with Nguyen and Stinglhamber (49), which suggest that to run the farming processes on a sustainable basis, it is necessary for the farmers that they must take care of the welfare of stakeholders like clients, suppliers, cow workers, and the public and develop good relations with them, irrespective of the size of farming. So, with high emotional labor, farmers adopt sustainable economic behaviors.

The study results revealed that mental health positively relates to sustainable economic behavior. These results agree with Miller et al. (51), which investigate the employees’ mental health role in farmer sustainable economic behavior. The study posits that the workers’ mental health determines their cognitive skills, physical abilities, decision-making, and behaviors. Farmers with good mental health, free from pressure, emotional distress, and financial worries, can have better cognitive skills, core physical abilities, and effective decision-making. Thus, they can adopt sustainable economic behavior as they can observe the environmental and social impacts of their functions and the activities of coworkers and take actions to mitigate negative impacts. It implies that if the farmer’s mental condition is not under the influence of any social distress, emotional disturbance, and financial distress and works effectively, they can better understand sustainable development. Thus, farmers with improved mental health can develop sustainable economic behavior.

The results indicated that mindfulness has a positive relation to emotional labor. These results are supported by Moon et al. (55), which sheds light on the farmer’s mindfulness and its role in emotional labor. The study shows that employees need to manage their emotions in the workplace. They must have to practice those emotions and behaviors that are required by the situation at a time. So, they need to keep changing their emotions with the change in the situation. It is possible only when the employees are fully present with all their senses and alertness at the workplace and observe the situation and respond to changes in the situation. It illumines that if workers have mindfulness, they can practice emotional labor. These results are also supported by Lee and Madera (56), which highlight that if the employees have mindfulness, they can better understand the situation, and they usually remain able to solve not only their own matters but also help their fellow workers. Thereby, they develop special bonding among themselves, and it becomes easy to practice emotional labor.

The results indicated that mindfulness has a positive relationship with mental health. These results are supported by Yang and Guy (57), which examine the role of mindfulness in improving mental health. The study implies that a man can have good mental health if he does not keep his mind idle, allowing it to get rusty, and does not keep it busy with just useless thoughts or activities. When a man has mindfulness, he lets his mind go through practice, paying attention to the situation around him and pondering on what is going on exactly, paying attention to someone’s utterances, and trying to think about what someone is saying and what has been the purpose behind the utterances, as well as analyzing his doings. So, he can improve his mental health. These results also agree with Bayighomog et al. (58), which reveal that in routine life, a man has to face many issues, and he may get the victim of emotional or mental distress. If he continues suffering from such distress, his mind may get paralyzed and stop working. Mindfulness motivates the man to keep on changing his thinking and not allow it just to stick to problematic matters. So, mindfulness helps maintain mental health.

The results revealed that emotional labor significantly mediators mindfulness and sustainable economic behavior. These results agree with Tran et al. (59), which reveal that when employees have mindfulness, they observe the situation around them, ponder its reasons, and find weak and good points. When they accept the changes and let the emotions change accordingly, they develop the ability of emotional labor. For making sustainable business decisions, the employees not only need to think through their minds rather have to be emotionally alert. So, as a result of mindfulness, emotional labor motivates employees to develop sustainable economic behavior. The results revealed that mental health significantly mediators mindfulness and sustainable economic behavior. These results agree with Flett et al. (60), which state that mindfulness helps employees in any economic sector, whether agriculture or industrial, to relieve emotional or financial distress and maintain mental health. This mental health assists in thinking that if they take care of the environmental and social welfare, they can have long-run economic progress.

## Implications

### Theoretical implications

This study has remarkable theoretical significance because it makes a lot of contributions to the literature on sustainable development. This study analyses the influences of mindfulness, emotional labor, and mental health on developing sustainable economic behavior in employees. In the previous studies, mindfulness, emotional labor, and mental health’s role in developing sustainable economic behavior in employees have been examined but with the help of a separate research survey. This study analyses these factors’ impacts on sustainable economic behavior. The relation of emotional labor and mental health to mindfulness and sustainable economic behavior has been researched. This article, which throws light on mediating role of emotional labor and mental health between mindfulness and sustainable economic behavior, removes the literary gap. This study is also a distinction, for it examines the influences of mindfulness, emotional labor, and mental health on developing sustainable economic behavior in employees at farms in China.

### Managerial implications

The agriculture sector of any country has tremendous significance from both social and economic points of view as it is the source of raw material, energy sources, and animal services to different economic sectors and food products, shelter, energy, and animal services to people living in society. However, unfortunately, this industry, like the others, also has environmental influences and is affecting the stakeholder’s welfare, and the negative performance of the farms from a social and environmental point of view restricts the long-term economic development. This article guides the policy-makers in establishing policies to improve farmers’ sustainable behavior by providing awareness, strong mental health, and emotional labor. This study has tremendous empirical significance to emerging economies like China, where agriculture is an important economic sector, and people rely much on this sector to meet their needs. This study provides a guideline for developing sustainable economic behavior among farmers. The study suggests that policies must be formed to develop mindfulness among the masses to develop sustainable economic behavior among them. The employees’ mindfulness must be aroused to improve their emotional labor and mental health, which can help develop sustainable economic behavior among employees.

## Conclusion

The study aims to examine the extent to which mindfulness, emotional labor, and mental health contribute toward sustainable economic behavior and to check the role of mindfulness in emotional labor and mental health. The study objective was also to analyze the role of emotional labor and mental health between mindfulness and sustainable economic behavior. The farmers in the agriculture sector of China were selected as study samples, and questionnaires were distributed to collect data for mindfulness, emotional labor, mental health, and sustainable economic behavior. The results revealed a positive relationship between mindfulness, emotional labor, and mental health to sustainable economic behavior. The results showed that when farmers have mindfulness, they pay attention to the influences of the firm’s activities on the environment, the natural resources (both living and non-living), and the welfare of stakeholders. Hence, it develops sustainable economic behavior when employees at agricultural farms can practice emotional labor. Likewise, the farmers’ mental health helps them develop thinking for sustainable economic development and adopt appropriate behavior while performing their duties at the workplace. Moreover, the study shows a positive link between mindfulness and emotional labor and mental health and a mediating role between mindfulness and sustainable economic behavior. Emotional labor and mental health are improved by mindfulness and further assist in developing sustainable economic behavior.

## Limitations and future research

Like many previous studies, this study also has several limitations. With intellectual expertise, the authors can remove these limitations and write a better study on sustainable economic behavior. The study addresses only three factors, mindfulness, emotional labor, and mental health, as indicators of developing sustainable economic behavior. Many other factors can help develop sustainable economic behavior in employees, like human resources management, organizational culture, and green investment. The data for mindfulness, emotional labor, mental health, and sustainable economic behavior and their relationship were collected from farmers in the agriculture sector of china. The Chinese agriculture sector has specific management and regulations, the economic conditions are particular in themselves, and the farming size is also different from that of the agriculture sector in other countries. So, this study may not be valid equally in all countries. Moreover, this study checks the mediating role of emotional labor and mental health between mindfulness and sustainable economic behavior. Since emotional labor and mental health also affect mindfulness and sustainable economic behavior, which can be used as moderators between mindfulness and sustainable economic behavior. So, it is recommended to future authors that they must take these factors as moderators between mindfulness and sustainable economic behavior in future studies.

## Data availability statement

The original contributions presented in this study are included in the article/supplementary material, further inquiries can be directed to the corresponding author.

## Ethics statement

Liaoning Petrochemical University, China reviewed and approved the studies involving human participants. The patients/participants provided their written informed consent to participate in this study. The study was conducted following the Declaration of Helsinki.

## Author contributions

YZ and ML: conceptualization and data collection. YQ: writing the draft. All authors agreed to the submitted version of the manuscript.
